# Data compression for sequencing data

**DOI:** 10.1186/1748-7188-8-25

**Published:** 2013-11-19

**Authors:** Sebastian Deorowicz, Szymon Grabowski

**Affiliations:** 1 Institute of Informatics, Silesian University of Technology, Gliwice, Poland; 2 Institute of Applied Computer Science, Lodz University of Technology, Łódź, Poland

## Abstract

Post-Sanger sequencing methods produce tons of data, and there is a general agreement that the challenge to store and process them must be addressed with data compression. In this review we first answer the question “why compression” in a quantitative manner. Then we also answer the questions “what” and “how”, by sketching the fundamental compression ideas, describing the main sequencing data types and formats, and comparing the specialized compression algorithms and tools. Finally, we go back to the question “why compression” and give other, perhaps surprising answers, demonstrating the pervasiveness of data compression techniques in computational biology.

## Background

In the first decade of the century, the cost of sequencing a single human genome fell from about 30 million to about 10 thousand dollars (here and later we mean U.S. dollars). The second generation sequencing platforms by 454 Life Sciences, Illumina, and Applied Biosystems cost less than one million dollars [1] and are available in many institutes. The promised third generation (3G) technology (including Ion Torrent Systems, Oxford Nanopore Technologies, and Pacific Biosciences equipment) should be even cheaper, which rapidly moves us closer to the day of personalized medicine available to the masses.

In mid-2013, the world-biggest sequencing institute, Beijing Genomics Institute, used 188 sequencers, of which 139 were the top-of-the-line Illumina HiSeq 2000 and 2500 sequencing machines. Their total theoretical throughput is over 1.2 Pbases per year, which is equivalent to about 3 PB of raw sequencing read files. Including the additional output space for mapping to the reference genomes, the total amount of necessary storage is on the order of 10 PB per year. The statistics of high-throughput sequencers in the world (http://omicsmaps.com) show that the storage necessary for the instruments’ output is in the range 50–100 PB per year. Kahn [2] presents the genomic data growth until 2010, pointing out that the progress in computer hardware lags behind.

Interestingly, recently the Million Veteran Program (MVP), led by the US Department of Veterans Affairs, was announced. With at least 30-fold coverage (100 bp reads) the number of reads per genome sample will be about 1 billion [3]. This means about 250 PB of raw data (in FASTQ format) in total, when the sequencing program is finished (the enrollment of volunteers is expected to last 5 to 7 years).

**Figure 1:**
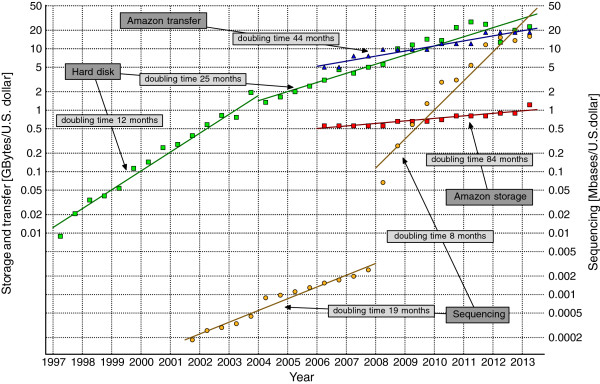
**Trends in storage, transfer, and sequencing costs.** The historic costs of low-end hard disk drives were taken from http://www.jcmit.com/diskprice.htm. They had been halving every 12 months in the 1990s and around 2000–2004. Then, the doubling time lengthened suddenly, to about 25 months. The real costs of sequencing, taken from the NHGRI Web page [5], reflect not only reagent costs like some studies show, but also include labor, administration, amortization of sequencing instruments, submission of data to a public database, etc. The significant change in sequencing costs around 2008 was caused by the popularization of the second generation technologies. The prices of the Amazon storage and transfer reflect the real market offers from the top data centers. It is interesting that the storage costs at data centers drop very slowly, mainly because the costs of blank hard disks are only a part of the total costs of maintenance. The curves were not corrected for inflation.

Those numbers are very large, but we need to remember that they refer to July of 2013. As can be seen in Figure 1, the cost of sequencing a single base has been halving roughly every 8 months in 2008–2013, while the cost of hard disk space has been halving every 25 months in 2004–2013. Even if the most recent NHGRI data suggest some stagnation (see also the comment [4]), it may be a temporary slowdown, as 3G instruments are becoming available (PacBio RS and Heliscope).

On the other hand, the prices of low-end hard disks are rather misleading, since the amount of data necessary in sequencing projects is so huge that data centers are much better places to store the files. The growing popularity of cloud storage can be attributed to several reasons. Generally, data management may be cheaper if run by IT professionals (not always available in smaller centers, like hospitals), centralization may reduce data replication costs, and access to really huge repositories (at least of terabyte scale) is easier or even only possible with large arrays of disks. The disk drives in large storage arrays are usually enterprise-class ones of increased reliability and better performance, so they are several times more expensive than standard (SATA) HD drives. Taking these factors together we should not be surprised that storing a file in a large data center may cost about 5–10 times more than on a plain HD; this is the price we pay for ubiquitous access to reliable data collections.

What is more, these files must be transferred over the Internet, which is not free. One of the largest data centers and cloud computing centers are Amazon S3 and Amazon EC2. Their charges for storage has been halving every 84 months and the charges for transfer has been halving every 44 months in 2006–2013. In January 2013 the real cost of sequencing human-size genome (according to NHGRI data) was about 5,700 dollars, while the cost of one-year storage at Amazon S3 and 15 downloads of raw reads and mapping results (225 GB of reads with 30-fold coverage and 500 GB of mapped data) was close to 1,500 dollars^a^.

The trends show clearly that the costs of storage and transfer will become comparable to the costs of sequencing soon, and the IT costs will be a significant obstacle for personalized medicine, if we do not face this problem seriously.

When choosing the compression algorithm to apply (if any), one should consider the specific use of the data. Two, in a sense extreme, scenarios are: (*i*) the data are to be transferred over a network (and then decompressed), (*ii*) the data are to be accessed in real-time, either in a sequential or random fashion. Let us take a closer look at both. In the case (*i*), a reasonable cost measure is the time to compress the given data (preferably using some “standard” computer), transfer it, and decompress on the receiver side. Concerning sequencing reads (FASTQ), it is possible to obtain (at least) 4.5-fold compression at about 30 MB/s both compression and decompression speed, on a single CPU core. Let us assume a link speed of 50 Mbit/s and that the input files have 450 GB in total. Without any compression the files will be transferred in exactly 20 hours. On the other hand, the compression, transfer and decompression times sum up to about 10.5 hours. Even if a faster connection is available (when the gain diminishes), we note that the compression and decompression may also be sped up (using threads or separate processes to utilize multiple CPU cores, and also by overlapping the compression, transfer and decompression phases). We point out that the ratio of 4.5, used in this example, is rather modest; significantly more is possible with lossy compression and also for some other biological data types (like genome collections).

Let us now estimate the monetary savings for transferring (downloading) these files from Amazon S3. The transfer charge depends on the volume of data, but let us assume 9 cents/GB. Without compression, we will pay 40.5 dollars for downloading these 450 GB of data, but only 9 dollars if compression is applied.

The case (*ii*) is somewhat different, as the choice of the compression method is rather constrained. To our knowledge, the most successful, in terms of efficiency and flexibility, storage system for biosequences was presented by Steinbiss and Kurtz [6]. Their GtEncseq solution accepts several common sequence formats (FASTA, GenBank, EMBL, FASTQ), compresses the input, provides fast random access to FASTA data, stores metadata, etc. Accessing single bases from random locations is not much slower than from the uncompressed equivalent representation, with over 3 million queries per second. In case of FASTQ, however, extracting a single read from an arbitrary location is not that fast, as it takes, e.g., more than 100 *μ*s, which is less than the access time of a hard disk drive, but not necessarily faster than the SSD. A space-time tradeoff exists, so this time may be reduced, but consequently compression ratio drops quite severely. Anyway, if only sequential access to FASTQ is needed (which is often the case), GtEncseq may be a good choice.

As new large-scale sequencing projects are frequently announced (the mentioned MVP project is probably the most ambitious at the moment), the storage concerns are of high priority. It is no wonder then that many ideas of selective storage (or lossy compression) are discussed in the community (cf. [7,8]). While we agree that some forms of lossy compression seem to be a necessity, the radical approach of discarding old data (in the hope of reproducing them later, when needed) raises major methodological doubts, as far as research applications are considered. More precisely, discarding raw data is hazardous, since their reproduction later will not be exact, due to inherent “randomness” in sequencing process, even if the same hardware and possibly identical procedure are used. This clashes with one of the main principles of the scientific method, which is reproducibility.

The area of data compression techniques in computational biology has been surveyed by Giancarlo *et al.*[9,10], with more focus on the theory and data compression applications in sequence analysis than storage and indexing of data from high-throughput technologies. One aspect of data compression in genomics, index structures for sequencing data, is thoroughly discussed by Vyverman *et al.*[11]. We hope our review complements earlier efforts by paying attention to versatile applications of data compression in the age of data flood in bioinformatics.

The paper is organized as follows. The next section contains a brief introduction to widely used data compression algorithms. The section “Sequencing data” outlines the popular compression techniques and file formats to store various bioinformatics data: base calls annotated with quality scores, genome alignment data, single and multiple genome data. Making data smaller is not only for reducing their space and facilitating their distribution, as it is reflected in the name of the next section, “Beyond storage and transfer”. Here, applications of data compression ideas in indexing, read alignment and other vital problems are discussed. The last section concludes.

### Data compression in brief

Compression techniques are the traditional means of handling huge data. Those methods reduce the space for storage and speed up the data circulation (e.g., among research institutes). With its origins yet in the 19th century and first theoretical works just after the WW2, nowadays data compression is used almost everywhere as the amount of stored and transmitted data is huge. There are several major concepts that are used in compression programs, but the two mentioned below are the most important in bioinformatics. Here we give only a very short description of them, with more details in the Additional file 1 or the monograph [12].

The Huffman coding [12,13], invented in 1952, is a statistical method, which assigns a sequence of bits (a *codeword*) to each alphabet symbol. The codewords are of different length, and in accordance to the golden rule of data compression, rarer symbols are represented by longer codewords. The given sequence is then encoded by replacing each symbol with its corresponding codeword. What makes Huffman coding important is its optimality, i.e., no other code leads to a shorter encoded sequence.

In 1977–78 Ziv and Lempel [12,14] invented dictionary methods. They process the sequence from left to right and encode possibly long repetitions of consecutive symbols as references to the already compressed part of data. Such an approach allows for higher compression ratios than sole Huffman coding as it looks for another type of redundancy, common not only in natural language (e.g., repeating word phrases), but also in multiple genome sequences or overlapping sequencing reads. Even better results are possible with combining dictionary methods and Huffman coding. The very popular gzip program serves as a successful example.

The Burrows–Wheeler transform (BWT) [12,15] is a more recent compression idea, that has become highly popular in bioinformatics. The pure BWT is not a compression method, it only permutes the input sequence, but it can be used to construct highly efficient compressors (bzip2, combining the BWT with Huffman coding and other techniques, is a well-known representative). This transform is also a basis of some sequence indexing techniques that are used to search a genome in many tools (more on this will be covered in the “Beyond …” section). The key idea of BWT is to permute the input sequence in such a way that symbols are grouped by their neighborhood, i.e., the symbols that are followed by the same symbols are close after the permutation, even if they were far in the original sequence (see also the Additional file 1 for a BWT example).

### Sequencing data

#### ***Raw sequencing data***

The raw reads from sequencing are usually stored as records in textual FASTQ format. Each record is composed of a read ID, base calls, and quality scores for all base calls [16]. These files are often compressed with gzip, to obtain about 3-fold size reduction. While significant, such a gain is not quite satisfactory. A better choice is to use a FASTQ-dedicated compressor, e.g., DSRC [17], which shrinks the data about 5 times. The algorithm finds overlaps in the base calls from the reads placed at very long distances in the file (in Ziv–Lempel fashion), as well as takes care of various ID format conventions. It also uses multiple statistical models to better compress quality values by the Huffman coding. In particular, quality scores in different read positions are compressed in different models (i.e., based on different statistics) because it is well-known that the quality deteriorates towards the read end, hence this design improves the prediction of the scores. DSRC is quite fast, as it compresses at 30–40 MB/s speed, and handles alphabets of size beyond 5 (IUPAC ambiguity codes) as well as variable-length reads.

Most newer proposals [18,19] focus mainly on compression ratio and either the speeds are of secondary importance or are not examined at all. For example, the solution by Bhola *et al.*[18] follows essentially the same direction as DSRC, but handles also approximate and palindromic repeats. The byproducts are compressed with an adaptive arithmetic coder [12]. While the reported compression ratios are usually higher than the DSRC’s by a few percent, it is unclear if the solution can be scalable. Remarkably, doubts are expressed by the authors themselves.

A more radical attempt to improve compression ratio is to group the reads with suffix-prefix overlaps close together. Unfortunately, the algorithms following this line [20,21] are not fully functional tools, e.g., they ignore the read IDs and quality scores, and achieve processing speeds on the order of 1 MB/s or less. A more interesting solution along these lines, SCALCE [22], is a FASTQ preprocessor helping to improve the compression ratio with gzip twice.

As argued earlier, the rapid growth of data from sequencing experiments demands even better compression ratios, and switching to a lossy mode seems to be the only chance for a breakthrough [23]. The natural candidates for lossy compression are quality scores. It has been shown [22,24-26] that rounding the quality scores to a few values (instead of about 60) can be acceptable. E.g., the fraction of discrepant SNPs grows slowly with diminishing number of quality scores in Illumina’s CASAVA package [27] while the benefit in compression is clear^b^. On the other hand, some (re)assemblers ignore these data [23], so if the reads are to be processed by such tools, the quality scores can be removed. Another option is to use the scores to prefilter the reads and discard the ones with unacceptably low quality.

SeqDB [28] is a notable exception from the shown tendency. Here the focus was put on compression and decompression speed, not compression ratio. On a machine with 12 CPU cores SeqDB reaches the speeds of about 500–600 MB/s ([28], Section 4.4), but the compression ratio is at best at gzip’s level.

Recently, two very interesting algorithms were presented. One of them is Quip [29], which uses higher-order modelling [12] with arithmetic coding in its more “traditional” mode, and also an assembly-based approach in its stronger mode. The idea is to form contigs from the first (by default 2.5 million) reads, which then are used as a reference for the following reads. The compression improvement due to this idea is moderate however, so the standard mode (faster and using half of the memory) seems to be more practical, with compression significantly better than DSRC and 10 MB/s to 20 MB/s compression/ decompression speed.

In [30] two FASTQ compressors were presented. Now we briefly describe the more interesting of them, Fqzcomp. It achieves better compression ratios thanks to a carefully chosen context mixing model^c^ and other original ideas. Fqzcomp belongs also to faster solutions, (partly) due to multi-threading. In the same paper, yet another strong FASTQ compressor was tested, SeqSqueeze1 (described only in [30]). This algorithm, also based on context mixing, is sometimes even better than Fqzcomp in respect of compression ratio, but its compression and decompression speed is less than 1 MB/s.

In Table 1 we compare the existing tools for compressing sequencing data. They belong to four different kinds described in the following four subsections. We did our best not to miss any relevant tool for which the sources or (at least) binary executables for a popular operating systems were available. There are several reasons why we do not present experimental (comparative) tests of the presented tools. They are, in the order of decreasing importance: 

 (*i*)limitations of many tools (e.g., accepting sequences with only the ACGT or only ACGTN alphabet, support for fixed-width reads only, assumptions on the ID format in FASTQ files, restriction to only selected fields of the SAM format);

 (*ii*) significant problems with running some of the existing tools (which we have experienced in earlier work, on FASTQ and genome collection compression);

 (*iii*) not quite compatible outputs (e.g., DSRC for FASTQ supports random access, which cannot be turned off, while many others do not, hence comparing compression ratios of these tools cannot be fully fair);

 (*iv*) different targets (by design, some of the tools are supposed to be run on a commodity PC, while others require powerful servers, or never were tested on mammalian-size data, since it would take days or even weeks. For example, many top single genome sequence compressors work at the rate of several Kbase/s only, on a standard computer).

**Table 1:** Summary of the most important compressors of sequencing data

**Software**	**Implementation**	**Website**	**Lossless /**	**Ambig.**	**Var.**	**Speed of**	**Ratio**	**Random**	**Methods**	**Remarks**
**name**	**availability**		**lossy**	**codes**	**length**	**compr./**		**access**		
	**src code / binaries / libs**				**reads**	**decompr.**				
**Compressors of raw sequencing data**										
gzip	C++ / many / many	http://www.gzip.org	yes / no	yes	yes	moderate / very high	low	no	LZ, Huf	
bzip2	C / many / many	http://www.bzip.org	yes / no	yes	yes	low / high	low	no	BWT, Huf	
7zip	C, C++ / many / many	http://www.7-zip.org	yes / no	yes	yes	low / very high	moderate	no	LZ, AC	
BWT-SAP [21]	C++ / — / C++	http://github.com/BEETL/BEETL	yes / no	yes	no	low / low	moderate	no	BWT, PPM	FASTA only
DSRC [17]	C++ / Lin, Win / C++, Pyt	http://sun.aei.polsl.pl/dsrc	yes / no	yes	yes	high / high	moderate	yes	LZ, Huf	
Fqzcomp [30]	C / Lin / —	http://sourceforge.net/projects/fqzcomp/	yes / yes	no	yes	high / moderate	high	no	CM	
G-SQZ [31]	C++ / Lin / —	http://public.tgen.org/sqz	yes / no	no	no	high / moderate	low	yes	Huf	
Kung-FQ [19]	C# / Win / –	http://quicktsaf.sourceforge.net	yes / yes	no	no	moderate / moderate	moderate	no	AC, LZ, RLE	
Quip [29]	C / – / –	http://cs.washington.edu/homes/dcjones/quip	yes / no	no	no	high / high	high	no	M. models, AC	
ReCoil [20]	C++ / — / C++	http://github.com/BEETL/BEETL	yes / no	no	no	very low / high	moderate	no	BWT, PPM	FASTA only
SCALCE+gzip [22]	C++ / – / –	http://scalce.sourceforge.net	yes / yes	no	no	moderate / high	moderate	no	AC, LZ, Huf	
Seq-DB [28]	C++ / – / –	https://bitbucket.org/mhowison/seqdb	yes / yes	no	no	very high / very high	low	yes	AC, LZ, RLE	
SeqSqueeze1 [30]	C/ Lin/ —	http://sourceforge.net/p/ieetaseqsqueeze/wiki/Home/	yes / no	no	yes	very low / ver low	high	no	CM	
**Compressors of reference genome alignment data**										
gzip	C++ / many / many	http://www.gzip.org	yes / no	yes	N/A	low / very high	low	no	LZ, Huf	
bzip2	C / many / many	http://www.bzip.org	yes / no	yes	N/A	low / high	low	no	BWT, Huf	
7z	C, C++ / many / many	http://www.7-zip.org	yes / no	yes	N/A	low / very high	moderate	no	LZ, AC	
BAM [32]	C++ / many / many	http://samtools.sourceforge.net	yes / no	yes	N/A	moderate / high	moderate	yes	LZ, Huf	
CRAM [33]	Java / many / Java	http://www.ebi.ac.uk/ena/about/cram_toolkit	yes / yes	yes	N/A	moderate / moderate	moderate	yes	Huf, Gol, diff.	
Quip [29]	C / – / –	http://cs.washington.edu/homes/dcjones/quip	yes / no	no	N/A	high / high	high	no	M. models, AC	
SAMZIP+rar [34]	C/ – / –	http://www.plosone.org/article/info: doi/10.1371/journal.pone.0028251	yes / no	yes	N/A	moderate / high	moderate	no	RLE, LZ, Huf	
**Compressors of single genome sequences**										
gzip	C++ / many / many	http://www.gzip.org	yes / no	yes	N/A	moderate / very high	low	no	LZ, Huf	
bzip2	C / many / many	http://www.bzip.org	yes / no	yes	N/A	low / high	low	no	BWT, Huf	
7z	C, C++ / many / many	http://www.7-zip.org	yes / no	yes	N/A	low / very high	moderate	no	LZ, AC	
dna3 [35]	C / – / –	http://people.unipmn.it/manzini/dnacorpus/	yes / no	no	N/A	low / low	moderate	no	LZ, PPM	
FCM-M [36]	C / – / –	http://ftp://www.ieeta.pt/~ap/codecs/	yes / no	no	N/A	very low / very low	moderate	no	M. models	
XM [37]	Java / many / Java	http://ftp.infotech.monash.edu.au/software/DNAcompress-XM	yes / no	yes	N/A	very low / very low	moderate	no	M. models, AC	
**Compressors of genome collections**										
gzip	C++ / many / many	http://www.gzip.org	yes / no	yes	N/A	low / very high	very low	no	LZ, Huf	
bzip2	C / many / many	http://www.bzip.org	yes / no	yes	N/A	low / high	very low	no	BWT, Huf	
7z	C, C++ / many / many	http://www.7-zip.org	yes / no	yes	N/A	low / very high	high	no	LZ, AC	chr-ordered
ABRC [38]	C++ / Lin, Win / C++	http://www2.informatik.hu-berlin.de/~wandelt/blockcompression/	yes / no	yes	N/A	high / very high	very high	yes	LZ, Huf	
GDC [39]	C++ / Lin, Win / C++	http://sun.aei.polsl.pl/gdc	yes / no	yes	N/A	high / very high	very high	yes	LZ, Huf	
GReEn [40]	C / – / –	http://ftp://ftp.ieeta.pt/~ap/codecs/	yes / no	yes	N/A	high / high	high	no	M. models, AC	
GRS [41]	C / Lin / –	http://gmdd.shgmo.org/Computational-Biology/GRS/	yes / no	yes	N/A	moderate / low	high	no	LCS, Huf	
RLZ [42]	C++ / – / –	http://www.genomics.csse.unimelb.edu.au/product-rlz.php	yes / no	yes	N/A	moderate / very high	high	no	LZ, Gol	

Abbreviations used in the table: src—source codes, libs—libraries, Lin—Linux, Win—Windows, Pyt—Python, exe—binary executables, AC—arithmetic coding (a statistical coding method [12]), CM—context mixing for arithmetic coding [12], diff—differential coding (paradigm: store only changes between sequences), Gol—Golomb (a statistical coding method [12]), Huf—Huffman, LCS—longest common subsequence (a measure of similarity of sequences [43]), LZ—an algorithm from Ziv–Lempel family, M. models—Markov models [12], PPM—prediction by partial matching (an efficient general-purpose compressor [12]). “Ambig. codes” means the ability to compress DNA symbols other than {*A, C, G, T, N* }. “chr-ordered” for 7z and genome collections means that the input (human) genomes were split into chromosomes and ordered according to them before the actual compression. In this way several chromosomes fit the 7z LZ-buffer which is beneficial for the compression.

#### ***Reference genome alignment data***

The reads are usually assembled or reassembled. *De novo* assembling is the most challenging, but resequencing, in which the reads are aligned to some reference genome, is much cheaper and thus widely used.

The results of mapping the reads onto the reference genome are usually stored in the SAM/BAM format [32]. SAM files augment the reads data with mapping quality and several other fields. BAM is a gzip-like compressed binary equivalent of textual SAM and is about 3–4 times smaller. Due to the additional data SAMs are more than twice as large as FASTQ files.

The reads in SAM files are mapped to a known reference genome and the differences between the reads and the reference sequence, resulting from variation and sequencing errors, are small. Thus, it is efficient to represent the base calls of a read as the mapping coordinate and the differences. These reads are usually ordered by the mapping coordinate and thus the coordinates can be stored in a differential manner, which results in a sequence of small and thus well compressible numbers. The oldest scheme [44] for compressing mapping data with the described idea cannot however be considered mature, since quality scores are ignored and there is no support for unaligned (i.e., those that failed to map onto a reference) reads.

Fritz *et al.*[33] handle both aligned and unaligned reads. The aligned reads are stored basically as described above together with the quality scores. To obtain better compression ratios, the authors advocate using a lossy mode and refrain from storing some quality scores, e.g., the ones related to perfectly matched positions. To compress the unaligned reads (usually 10–40% of raw reads) better, they propose to build some artificial reference sequences. To this end, unmapped reads from many similar experiments are processed by an assembler to obtain contigs, built only for the compression process. Finally, the remaining sequences are matched to the bacterial and viral databases. Some of the byproducts in the algorithm are encoded with the Huffman (or other) codes. Most of the described techniques, excluding artificial reference as well as bacteria and viral sequences, are implemented in the CRAM compressor. In a highly lossy setting, it can produce archives smaller by an order of magnitude than corresponding BAM files. Similar approaches for compressing reads by mapping them onto an reference genome are used in SlimGene [25] and SAMZIP [34] algorithms.

Generally the use of a reference sequence can help a lot for the compression ratio but we should remember that a reference might not be available in some cases, e.g., for metagenomic datasets or for organisms with high polymorphism [20].

The most recent SAM compressor [45], apart from highly configurable lossy compression settings, introduces a novel idea to exploit common features of reads mapped to the same genomic positions. Quip [29], published slightly earlier, is not as flexible as CRAM and works only losslessly. However, if aligned reads in the SAM or BAM format and a reference sequence are given, it wins with CRAM in compression ratio and needs less memory to operate.

The tabix program [46] is a more general solution, popular in sequencing centers. It was designed to allow fast random access to compressed (gzip-like) textual files, in which the data are stored in rows containing tab-delimited values. The basic idea is to sort the input rows according to the sequence name and coordinate. Then the file is split into a series of blocks of maximum size of 64 KB. These blocks are compressed. Finally, an index to support random access queries is built.

#### ***Single genome sequences***

Raw and annotated sequencing data are nowadays the greatest challenge for storage and transfer today. Nevertheless, consensus DNA sequences (e.g., complete bacterial genomes) used to be historically the first object of compression in bioinformatics. In a sense, however, the genome sequences for a single individual are almost incompressible. If the sequence contains only the symbols A, C, G, and T, then the trivial 2 bits per symbol encoding is often more efficient than a general-purpose compressor, like gzip!

Specialized DNA compressors appeared in mid-1990s, but most solutions from the literature are impractically slow. For example, one of the strongest of them, the highly-acclaimed XM [37], can squeeze a genome up to about 5 times, but compression speed of the order of 20 KB/s on a modern machine is clearly disappointing. Some other notable compressors in this area are dna3 [35] and FCM-M [36]. The standard input format for genome sequences is FASTA, in which the file starts with a single-line description, followed by lines of sequence data.

#### ***Collections of genome sequences***

As said, a single genome in its compact encoding (2 bits per base) seems almost incompressible. However, large repositories with thousands of individual genomes of the same species are just behind the corner. These genomes are highly similar to each other (e.g., human genomes have more than 99% of their content in common [47]), so a collection can be very efficiently compressed. Dictionary methods, from the LZ family, constitute the most obvious and actually most successful approach, considering high-speed decompression with moderate memory requirements. The compression phase is more demanding, both in time and space, because the repetitions (matches) in a collection of genomes are typically gigabytes apart. For these reasons, most general-purpose LZ-style compressors (e.g., gzip, rar) are useless for those data, and a few years ago the first specialized algorithms emerged.

In their seminal work, Christley *et al.*[48] compressed a single human (James Watson’s) genome, but with the variation data relative to a reference genome being provided. Variation data were comprised of single nucleotide polymorphisms (SNPs) and so-called indels, i.e., insertions or deletions of multiple nucleotides. Additionally, they used a readily-available SNP database. The assumed scenario, augmented with standard compression techniques, made it possible to represent the whole human genome in about 4 Mbytes only. Recently, Pavlichin *et al.*[49] followed the lines pioneered in [48], compressing the JW genome to 2.5 MB, with very similar average results on 1092 human genomes from the 1000 Genomes Project. The introduced novelties are partly biologically-inspired, e.g., making use of the tag SNPs characterizing haplotypes. In another recent achievement, Deorowicz *et al.*[50] compressed the variant call files from 1092 diploid *H. sapiens* (1000 Genomes Project) and 775 *A. thaliana* (1001 Genomes Project) individuals, together with a variant database, squeezing the former collection to about 432 MB and the latter to 110 MB (which translates to 395 KB and 142 KB per individual, respectively).

Other research did not assume access to a knowledge base (i.e., a reference genome), hence most of them did not yet achieve the mentioned levels of compression. Most of these works [39,40,42,51] encode one of the genomes in a collection with simple means, spending about 2 bits per base, and then apply very efficient *differential* LZ-like encoding for the remaining genomes. The pioneering algorithm of this kind, RLZ [42,52], looks for LZ-matches in the reference genome (the one encoded naïvely, with 2 bits per base), and encodes their positions compactly, with reference to the previous match. This handles typical differences in genomes of the same species (short insertions or deletions, SNPs) in an efficient manner. Such an approach is successful: the related compressor, GDC [39], obtains in its strongest mode the compression ratio of 1,000 for relatively encoded genomes in a collection of 70 human individuals, with decompression speed of 150 MB/s. The key ideas in GDC include looking for long approximate matches in the whole collection and the Huffman coding.

Some of the specialized compressors (GDC, LZ-End [53]) allow for fast access to an arbitrary substring of the compressed collection. Unfortunately, this comes at a price: some loss in compression ratio (which still remains competitive, though).

Yet another differential genome compressor was presented by Wandelt and Leser [38]. Implementation-wise its original trait is searching for matches via a compressed suffix tree [54] (in blocks). This solution reaches the compression ratio of about 400 for the collection of 1000 human genomes. The match-finding speed of a parallel implementation is rather high (85 MB/s with large blocks). The real bottleneck is, however, building the compressed suffix tree.

Interestingly, also 7zip, a well-known advanced general-purpose LZ compressor, achieves quite competitive results, but for mammalian-size genomes the collections must be reordered by chromosomes, otherwise it cannot find inter-genome LZ-matches and its compression then is not much better than gzip’s. This behavior can be explained by its LZ-buffer limited to 1 GB, which is less than the size of, e.g., the human genome. On the other hand, chromosomes are already small enough and several of them fit its buffer.

Currently, public repositories often store large genomes as variant databases (e.g., in VCF format). This tendency should help significantly in developing new efficient compression algorithms, since all input sequences are perfectly aligned and the (otherwise hard and resource-consuming) task of finding repetitions in data becomes almost trivial. Thus, we expect the work by Christley *et al.*[48], Pavlichin *et al.*[49] and Deorowicz *et al.*[50] to be only the first steps in this direction.

### Beyond storage and transfer

So far, we have discussed how compression can alleviate the burden of storing and transmitting various genomic data. It can, however, help in less obvious aspects as well.

One prominent example is *de novo* assembly for second-generation sequencing technology, based on the de Bruijn graph [55], where hundreds of GB of RAM could be needed with standard methods. Applying succinct data structures allowed to decrease the memory usage by an order of magnitude, and a whole assembly pipeline of a human individual was run in about 36 GB of RAM [56]. The nodes in the de Bruijn assembly graph are all distinct strings of some length *k* (e.g., 25) from given data, and edges between them exist if two nodes have a suffix-prefix overlap of length *k*-1. The idea of Conway and Bromage [56] was to perceive the set of edges for given data as a subset of the full graph and to use (existing) succinct subset encoding techniques, supporting fast access. Representing this subset with naïve means would be impossible because of typically quadrillion-edge scale of the full graph. On the other hand, standard approaches to the de Bruijn assembly graph construction are plagued with pointers (location addresses in the memory), which are dominant part of the data structure in RAM.

Compact data structures not always make use of “typical” compression techniques (like statistical coding or LZ-matches), yet they serve the same purpose, which is (in the currently discussed application) reducing the memory requirements for building or querying a large graph. The Bloom filters [57], the well-known idea for compact approximate subset representation, were recently used with success for the de Brujin graph construction [58,59]. In particular, the work of Salikhov *et al.*[59] is a refinement of the technique of Chikhi and Rizk [58]. Their graph built for 564M human reads of length 100 bp using *k*=23 occupies only about 2.5 GB. Another succinct (but not exactly compression) technique was proposed by Ye *et al.*[60]. They store only a small subset of the observed *k*-mers as nodes, with their neighboring chains of bases as edges. Some care is taken to remove low-coverage edges, which normally result in tips, loops or bubbles in the assembly graph, undesirable in the graph for a few reasons, including very high memory requirements. The compact structure of SparseAssembler from the cited work was built in less than 2 hours for the human chromosome 14 with the peak memory use of 3 GB. The memory requirement for the whole human genome was below 30 GB. While the result from [59], cited above, seems clearly better, they are not directly comparable, unfortunately, due to different coverages and used values of *k*.

An alternative to the de Bruijn graph is the assembly *string graph*[61], not working on *k*-mers, but requiring fast and memory efficient algorithms for the computation of suffix-prefix overlaps of arbitrary length among reads. In a string graph, as opposed to the de Bruijn graph, each path represents a valid assembly of the reads (because the reads are not “decomposed” into many independent *k*-mers). Although this approach seems harder, in the SGA string assembler [62] error correction and assembling were performed with satisfactory accuracy on 125 Gbp of human genome reads using 54 GB of memory. This was achieved thanks to a compressed data structure, the FM-index [63], which will be mentioned also later. Interestingly, another practical string graph assembler, Readjoiner [64], which can process 115 Gbp short reads dataset in 52 GB of RAM, does not make use of compressed data structures, but its space effectiveness comes from an ingenious partitioning approach applied to the array of a relevant subset of all suffixes of all reads. Readjoiner also confirms that compact data structures may be fast because of locality of accesses to data.

Another compression example concerns data *indexing*. Computational biology is mostly about data analysis, which in turn involves pattern search. If the data over which patterns are sought do not change over time, we talk about a static scenario. This is quite common, e.g., an already sequenced genome of a given individual is usually not updated for a long period. In such a case it may be worth to build an index structure for given data since its construction time, even if significant, is likely to be paid off during multiple subsequent pattern searches. A classic text indexing data structure is the *suffix tree* (ST) [43]. It is powerful and useful, but unfortunately requires up to 28*n* bytes of space, where *n* is the sequence length. Thus, it is hard to store an ST in the main memory even for a single mammalian genome. The *compressed index* idea is to support all (or main) functionalities of its classic counterpart (e.g., returning the locations of all occurrences of the pattern in the text), but using much less space. The area of compressed indexes, initiated only around 2000, has been marked by tens of significant papers [65]. Unfortunately, those “general-purpose” text indexes are not a good choice for a collection of genomes of individuals of the same species. In these cases, LZ-based indexes are much more efficient in removing the specific (and very large) redundancy. Several works with LZ-style indexes designed for genomic data appeared in the recent years. Most of the solutions for the exact [66-68] and approximate pattern search [69] are rather theoretical as for only some of them implementations are available.

The Burrows–Wheeler transform (BWT) is used with huge success for mapping sequencing reads onto a reference genome, almost making the classic, *q*-gram-based approach, obsolete (for example, an interesting representative of the latter approach, Hobbes [70], is very fast but also uses large amount of memory). Some of the most important genome alignment algorithms, Bowtie [71,72], BWA [73], BWA-SW [74], SOAP2 [75], and GEM [76], make use of the FM-index [63] or another compressed index based on the BWT, occupying as little as about 2 GB for a human genome (with the exception of GEM, requiring usually from 3 GB to 6 GB). For more information on BWT and FM-index, see the Additional file 1. These aligners also belong to the fastest ones. All of them (Bowtie only in version 2) support ungapped and gapped alignments and all of them are multi-threaded to make use of multi-core CPUs. Using BWT for gapped alignment is cumbersome, and this is why Bowtie2 [72] and GEM combine it with dynamic programming, a classic computation technique boasting its flexibility and tolerance for large gaps and affine gap penalties. An important issue for compressed indexes is the working space needed during their construction, as standard BWT computation algorithms require at least 5*n* bytes of memory. Lightweight algorithms for BWT computation [77,78] appeared relatively late, yet the Kärkkäinen’s method [77] is already implemented in Bowtie.

A special case of mapping sequence reads to genomes concerns RNA-Seq experiments, in which a ‘snapshot’ of RNA molecules in the cell is sequenced. The RNA-Seq [79] is a relatively new approach that proved highly successful especially for determination of gene expression. The main problem here is that we must deal with reads at exon-exon boundaries (without the introns present in the reference genome), so spliced mappings must be looked for, which is unusual for standard DNA reads mapping. Some mappers for this problem also make use of the FM-index, e.g., TopHat [80] and CRAC [81]. A comprehensive list of RNA-Seq mapping approaches can be found in [82].

The FM-index searches for a pattern finding its successive letters, from right to left, which can be called backward extension of a string. Recently, Li [83] presented a simple modification of the FM-index for forward-backward extension of DNA strings. His *de novo* assembler, fermi, shows that the assembly based variant calling can achieve an SNP accuracy close to the standard mapping approach, being particularly strong in indel calling.

It is worth noting that BWT-based alignment can be implemented on a massively parallel graphics processing unit (GPU). Recent tools SOAP3 [84] and SOAP3-dp [85], being about an order of magnitude faster than their CPU-based counterparts, are prominent examples.

One could ask if the FM-index, or another compressed index, is useful for searching DNA strings in a genome, e.g., given as FASTA input. The answer is positive; thanks to the small alphabet the search times (the *count* query, in which we return the number of matches only) of the FM-index, in the best current implementation, may be comparable with the suffix array ([86], Table Six and Seven). The space use, however, is only about 0.3*n* (in contrast to 5*n* needed by the suffix array). On the other hand, the *locate* query, in which the positions of all matching substrings are returned, is at least an order of magnitude slower, and needs some extra space.

In some cases, however, searching directly in the compressed data may be faster than in the straightforward representation. Loh *et al. *[87] compress a sequence database so that if an inserted sequence is similar enough to one from the database, it is represented as the reference plus a list of differences (edit script). The search algorithm they propose, based on BLAST, takes care of the differentially encoded sequences, and only rarely requires to bring them back to their “full” form. Their Compressive BLAST / BLAT algorithm was found to be about 4 times faster than classic BLAST / BLAT tools [88,89]. Other examples where data compression reportedly speeds up processing concern the *k*-mer counting task, especially in I/O-constrained scenarios ([90], Table Two–Four). We note that reading compressed input is nowadays a convenient feature of many tools (e.g., *de novo* assemblers Velvet [91] and ABySS [92]), but not always it brings improvements in speed.

Compression methods were used also for other purposes, in which the goal was not the reduction of space or processing speed-up, but rather better understanding of genomic data. Cao *et al.*[93] used the XM algorithm [37] to align eukaryotic-size genomes in a few hours on a workstation. The idea is to teach the expert models on one of the sequences and use the knowledge to properly align the second one by measuring the information content and the mutual information content of the sequences.The resulting aligner is shown experimentally to be superior (at least in quality, not in speed) to conventional alignment methods based on character matching.

Bhaduri *et al. *[94] proposed a somewhat related idea of using a compression algorithm from the LZ family to filter low-complexity reads in a project on identification of nonhuman sequences, such as viruses, in deep sequencing datasets.

A measure of sequence similarity, that is both accurate and rapidly computable, is highly desirable. Ferragina *et al.*[95] advocated that classic alignment methods do not scale well for huge data. They focus on the Universal Similarity Measure (USM) [96]. As USM is rather a theoretical concept, the authors experiment with its three approximations based on data compression. They validate the possibility of using these approximations for classification of sequences by UPGMA and NJ methods.

Freschi and Bogliolo [97] proposed a lossy compression scheme to eliminate tandem repeats from a sequence. Thanks to that, no repeat masking is necessary before performing pairwise alignment of sequences.

### Conclusions

Data deluge in computational biology has become a fact. A vast majority of gathered data is “temporary” in nature and could be discarded as soon as the analysis is done. The problem is, however, that current sequence analysis algorithms are imperfect, and storing lots of data only in the hope to squeeze out more of them in the future is a reasonable strategy. To put it in other words, lossy storage is an interesting option for bioinformatics, but it should be used judiciously.

The variety of genomic data formats implies the need for specialized compression algorithms better than the general-purpose standards, like gzip and bzip2. Succinct representation is not everything; decompression time or rapid access to arbitrary data snippets may matter even more, so they should be taken into account in algorithmic design. Sometimes even more enhanced functionalities are welcome. Fast search directly in the compressed data is an example. More efficient compression diminishes the costs of not only local data storage and transfer, but also of data center services. The latter should bring the vision of ubiquitous cloud computing closer.

Let us make two predictions at the end. First, we note that some objects of interest in computational biology, like a human genome, do not grow. Hence, with growing amount of memory even in our home laptops, it perhaps no longer pays to apply strong compression for some tasks, if less compact but faster solutions are known. Read alignment onto a reference genome is a prominent example of this sort. We anticipate that in 1–2 years solutions processing a 1 billion 100 bp reads collection in a few hours on a PC will appear, but their main data structure may be the good old suffix array rather than, e.g., the FM-index.

Second, we predict that the turbulent period of new compression ideas for sequencing data representations will slowly give way to industry-oriented solutions, with more stress on robustness, flexibility, ease of use, and compression and decompression speed (in sequential and parallel/distributed regimes). Ideas are exiting, but routine jobs require standards. We believe that powerful, versatile and thus widely used formats in bioinformatics will emerge soon, proving the maturity of the field.

## Endnotes

^a^ There are, of course, many alternative cloud storage solutions and it is hard to tell “typical” fees for storage and transfer, as opposed to retail disk media prices which can be monitored rather easily. As a reference, however, we note that Microsoft Windows Azure and Google Cloud Storage charges in the same scenario are similar (about 1,350–1,500 dollars), and all these providers charge more for the assumed 15 downloads than for one-year storage.

^b^ Illumina software for their HiSeq 2500 equipment contains an option to reduce the number of quality scores [98]. Its effect on overall sequencing is shown in a technical support note [99].

^c^ Statistical methods often encode symbols with regard to the gathered statistics of occurrences in their respective *contexts*, which are formed with, e.g., several proceeding symbols. This approach can be made even more sophisticated with considering several contextual models running in parallel, in order to improve the estimation of symbols’ probability and, in result, the obtained compression ratio. The name of “context mixing” refers to this approach, in which the statistics from different contexts are “mixed” (weighted, blended).

## Competing interests

The authors declare that they have no competing interests.

## Supplementary Material

Additional file 1 Description of selected compression methods. Click here for file

## References

[B1] Metzker ML Sequencing technologies–the next generation Nat Rev Genet 2010 11 31 46 19997069 10.1038/nrg2626

[B2] Kahn SD On the future of genomic data Science 2011 331 728 729 21311016 10.1126/science.1197891

[B3] Roberts JP Million veterans sequenced Nat Biotechnol 2013 31 6 470

[B4] Hall N After the gold rush Genome Biol 2013 14 5 115 23657273 10.1186/gb-2013-14-5-115 PMC3663089

[B5] National Human Genome Research Institute, DNA Sequencing Costs [http://www.genome.gov/sequencingcosts/] (accessed February 14, 2013)

[B6] Steinbiss S Kurtz S A new efficient data structure for storage and retrieval of multiple biosequences IEEE/ACM Trans Comput Biol Bioinformatics 2012 9 2 345 357 10.1109/TCBB.2011.146 22084150

[B7] Kodama Y Shumway M Leinonen R The sequence read archive: explosive growth of sequencing data Nucleic Acids Res 2012 40 Database issue 54 56 10.1093/nar/gkr854 PMC3245110 22009675

[B8] Cochrane G Cook CE Birney E The future of DNA sequence archiving GigaScience 2012 1 1 article no. 2 10.1186/2047-217X-1-2 PMC3617450 23587147

[B9] Giancarlo R Scaturro D Utro F Textual data compression in computational biology: A synopsis Bioinformatics 2009 25 13 1575 1586 19251772 10.1093/bioinformatics/btp117

[B10] Giancarlo R Scaturro D Utro F Textual data compression in computational biology: Algorithmic techniques Comput Sci Rev 2012 6 1 1 25 10.1093/bioinformatics/btp117 19251772

[B11] Vyverman M De Baets B Fack V Dawyndt P Prospects and limitations of full-text index structures in genome analysis Nucleic Acids Res 2012 40 15 6993 7015 22584621 10.1093/nar/gks408 PMC3424560

[B12] Salomon D Motta G Handbook of data compression 2010 London: Springer

[B13] Huffman D A method for the construction of minimum-redundancy codes Proceedings of the Institute of Radio Engineers 1952 1098 1101

[B14] Ziv J Lempel A A universal algorithm for sequential data compression IEEE Trans Inf Theory 1977 IT-23 337 343

[B15] Burrows M Wheeler D A block sorting lossless data compression algorithm Technical Report 124, Digital Equipment Corporation 1994, http://www.hpl.hp.com/techreports/Compaq-DEC/SRC-RR-124.pdf.

[B16] Cock PJA Fields CJ Goto N Heuer ML Rive PM The Sanger FASTQ file format for sequences with quality scores, and the Solexa/Illumina FASTQ variants Nucleic Acids Res 2010 38 6 1767 1771 20015970 10.1093/nar/gkp1137 PMC2847217

[B17] Deorowicz S Grabowski Sz Compression of DNA sequence reads in FASTQ format Bioinformatics 2011 27 6 860 862 21252073 10.1093/bioinformatics/btr014

[B18] Bhola V Bopardikar AS Narayanan R Lee K Ahn T Wu F-X, Zaki M, Morishita S, Pan Y, Wong S, Christianson A, Hu X No-reference compression of genomic data stored in FASTQ format Proceedings of the IEEE International Conference on Bioinformatics and Biomedicine 2011 Atlanta, USA: IEEE Computer Society 147 150

[B19] Grassi E Di Gregorio F Molineris I KungFQ: A Simple and Powerful Approach to Compress Fastq Files IEEE/ACM Trans Comput Biol Bioinformatics 2012 9 6 1837 1842 10.1109/TCBB.2012.123 23221092

[B20] Yanovsky V ReCoil—an algorithm for compression of extremely large datasets of DNA data Algo Mol Biol 2011 6 23 10.1186/1748-7188-6-23 PMC3219593 21988957

[B21] Cox AJ Bauer MJ Jakobi T Rosone G Large-scale compression of genomic sequence databases with the Burrows-Wheeler transform Bioinformatics 2012 28 11 1415 1419 22556365 10.1093/bioinformatics/bts173

[B22] Hach F Numanagić I Alkan C Sahinapl SC SCALCE: boosting Sequence Compression Algorithms using Locally Consistent Encoding Bioinformatics 2012 28 23 3051 3057 23047557 10.1093/bioinformatics/bts593 PMC3509486

[B23] Miller JR Koren S Sutton G Assembly algorithms for next-generation sequencing data Genomics 2010 95 6 315 327 20211242 10.1016/j.ygeno.2010.03.001 PMC2874646

[B24] Wan R Anh VN Asai K Transformations for the compression of FASTQ quality scores of next generation sequencing data Bioinformatics 2011 28 5 628 635 22171329 10.1093/bioinformatics/btr689

[B25] Kozanitis C Saunders C Kruglyak S Bafna V Varghese G Compressing genomic sequence fragments using SlimGene J Comput Biol 2011 18 3 401 413 21385043 10.1089/cmb.2010.0253 PMC3123913

[B26] Ochoa I Asnani H Bharadia D Chowdhury M Weissman T Yona G QualComp: a new lossy compressor for quality scores based on rate distortion theory BMC Bioinformatics 2013 14 187 23758828 10.1186/1471-2105-14-187 PMC3698011

[B27] Illumina Casava v. 1.8.2 Documentation 2013 [http://support.illumina.com/sequencing/sequencing_software/casava.ilmn]

[B28] Howison M High-throughput compression of FASTQ data with SeqDB IEEE/ACM Trans Comput Biol Bioinformatics 2013 10 1 213 218 10.1109/TCBB.2012.160 23702558

[B29] Jones DC Ruzzo WL Peng X Katze MG Compression of next-generation sequencing reads aided by highly efficient de novo assembly Nucleic Acids Res 2012 40 22 e171 22904078 10.1093/nar/gks754 PMC3526293

[B30] Bonfield JK Mahoney MV Compression of FASTQ and SAM format sequencing data PLoS ONE 2013 8 3 e59190 23533605 10.1371/journal.pone.0059190 PMC3606433

[B31] Tembe W Lowey J Suh E G-SQZ: compact encoding of genomic sequence and quality data Bioinformatics 2010 26 17 2192 2194 20605925 10.1093/bioinformatics/btq346

[B32] Li H Handsaker B Wysoker A Fennell T Ruan J Homer N Marth Abecasis G Durbin R 1000 Genome Project Data Processing Subgroup The sequence alignment/map (SAM) format and SAMtools Bioinformatics 2009 25 16 2078 2079 19505943 10.1093/bioinformatics/btp352 PMC2723002

[B33] Fritz MH-Y Leinonen R Cochrane G Birney E Efficient storage of high throughput DNA sequencing data using reference-based compression Genome Res 2011 21 734 740 21245279 10.1101/gr.114819.110 PMC3083090

[B34] Sakib MN Tang J Zheng WJ Huang C-T Improving transmission efficiency of large sequence alignment/map (SAM) files PLoS ONE 2011 6 12 e28251 22164252 10.1371/journal.pone.0028251 PMC3229529

[B35] Manzini G Rastero M A simple and fast DNA compressor Softw Pract Exp 2004 34 14 1397 1411

[B36] Pinho AJ Ferreira PJSG Neves AJR Bastos CAC On the representability of complete genomes by multiple competing finite-context (Markov) models PLoS ONE 2011 6 6 e21588 21738720 10.1371/journal.pone.0021588 PMC3128062

[B37] Cao MD Dix TI Allison L Mears C A simple statistical algorithm for biological sequence compression Proceedings of the Data Compression Conference 2007 Washington, DC, USA: IEEE Computer Society Press 43 52

[B38] Wandelt S Leser U Adaptive efficient compression of genomes Algo Mol Biol 2012 7 30 10.1186/1748-7188-7-30 PMC3541066 23146997

[B39] Deorowicz S Grabowski Sz Robust relative compression of genomes with random access Bioinformatics 2011 27 11 2979 2986 21896510 10.1093/bioinformatics/btr505

[B40] Pinho AJ Pratas D Garcia SP GReEn: a tool for efficient compression of genome resequencing data Nucleic Acids Res 2012 40 4 e27 22139935 10.1093/nar/gkr1124 PMC3287168

[B41] Wang C Zhang D A novel compression tool for efficient storage of genome resequencing data Nucleic Acids Res 2011 39 7 e45 21266471 10.1093/nar/gkr009 PMC3074166

[B42] Kuruppu S Puglisi SJ Zobel J Reynolds M Optimized relative Lempel-Ziv compression of genomes Proceedings of the ACSC Australasian Computer Science Conference 2011 Sydney, Australia: Australian Computer Society, Inc. 91 98

[B43] Gusfield D Algorithms on strings, trees and sequences: Computer science and computational biology 1997 Cambridge, UK: Cambridge University Press

[B44] Daily K Rigor P Christley S Hie X Baldi P Data structures and compression algorithms for high-throughput sequencing technologies BMC Bioinformatics 2010 11 514 20946637 10.1186/1471-2105-11-514 PMC2964686

[B45] Popitsch N von Haeseler A NGC: lossless and lossy compression of aligned high-throughput sequencing data Nucleic Acids Res 2013 41 1 e27 23066097 10.1093/nar/gks939 PMC3592443

[B46] Li H Tabix: fast retrieval of sequence features from generic TAB-delimited files Bioinformatics 2011 27 5 718 719 21208982 10.1093/bioinformatics/btq671 PMC3042176

[B47] Levy S Sutton G Ng PC Feuk L Halpern AL Walenz BP Axelrod N Huang J Kirkness EF Denisov G Lin Y MacDonald JR Pang AWC Shago M Stockwell TB Tsiamouri A Bafna V Bansal V Kravitz SA Busam DA Beeson KY McIntosh TC Remington KA Abril JF Gill J Borman J Rogers YH Frazier ME Scherer SW Strausberg RL Venter JC The diploid genome sequence of an individual human PLoS Biol 2007 5 10 e254 17803354 10.1371/journal.pbio.0050254 PMC1964779

[B48] Christley S Lu Y Li C Xie X Human genomes as email attachments Bioinformatics 2009 25 2 274 275 18996942 10.1093/bioinformatics/btn582

[B49] Pavlichin D Weissman T Yona G The human genome contracts again Bioinformatics 2013 29 17 2199 2202 23793748 10.1093/bioinformatics/btt362

[B50] Deorowicz S Danek A Grabowski Sz Genome compression: a novel approach for large collections Bioinformatics 2013 29 20 2572 2578 23969136 10.1093/bioinformatics/btt460

[B51] Chern BG Ochoa I Manolakos A No A Venkat K Weissman T Reference based genome compression Publicly available preprint arXiv:1204.1912v1 2012

[B52] Kuruppu S Puglisi SJ Zobel J Chávez E, Lonardi S Relative Lempel–Ziv compression of genomes for large-scale storage and retrieval Proceedings of the 17th International Symposium on String Matching and Information Retrieval (SPIRE) 2010 Springer-Verlag, Berlin-Heidelberg: Springer, LNCS 6393 201 206

[B53] Kreft S Navarro G LZ77-like compression with fast random access Proceedings of the Data Compression Conference 2010 Washington, DC, USA: IEEE Computer Society 239 248

[B54] Ohlebusch E Fischer J Gog S Chávez E, Lonardi S CST++ Proceedings of the 17th International Symposium on String Matching and Information Retrieval (SPIRE) 2010 Springer-Verlag, Berlin-Heidelberg: Springer, LNCS 6393 322 333

[B55] Compeau PE Pevzner PA Tesler G How to apply de Bruijn graphs to genome assembly Nat Biotechnol 2011 29 11 987 991 22068540 10.1038/nbt.2023 PMC5531759

[B56] Conway TC Bromage AJ Succinct data structures for assembling large genomes Bioinformatics 2011 27 4 479 486 21245053 10.1093/bioinformatics/btq697

[B57] Bloom BH Space/time trade-offs in hash coding with allowable errors Commun ACM 1970 13 7 422 426

[B58] Chikhi R Rizk G Raphael BJ, Tang J Space-efficient and exact de Bruijn graph representation based on a Bloom filter Proceedings of the 12th International Workshop on Algorithms in Bioinformatics (WABI) 2012 Springer-Verlag, Berlin-Heidelberg: Springer, LNCS 7534 236 248

[B59] Salikhov K Sacomoto G Kucherov G Darling A. E., Stoye J Using cascading Bloom filters to improve the memory usage for de Brujin graphs Proceedings of the 13th International Workshop on Algorithms in Bioinformatics (WABI) 2013 Springer-Verlag, Berlin-Heidelberg: Springer, LNCS 8126 364 376

[B60] Ye C Ma ZS Cannon CH Pop M Yu DW Exploiting sparseness in de novo genome assembly BMC Bioinformatics 2012 13 Suppl 6 S1 22537038 10.1186/1471-2105-13-S6-S1 PMC3369186

[B61] Myers EW The fragment assembly string graph Bioinformatics 2005 21 suppl 2 ii79 ii85 16204131 10.1093/bioinformatics/bti1114

[B62] Simpson JT Durbin R Efficient de novo assembly of large genomes using compressed data structures Genome Res 2012 22 549 556 22156294 10.1101/gr.126953.111 PMC3290790

[B63] Ferragina P Manzini G Opportunistic data structures with applications Proceedings of the 41st Annual Symposium on Foundations of Computer Science (FOCS) 2000 Redondo Beach, California, USA: IEEE Computer Society 390 398

[B64] Gonnella G Kurtz S Readjoiner: a fast and memory efficient string graph-based sequence assembler BMC Bioinformatics 2012 13 82 22559072 10.1186/1471-2105-13-82 PMC3507659

[B65] Navarro G Mäkinen V Compressed full-text indexes ACM Computing Surv 2007 39 2

[B66] Kreft S Navarro G On compressing and indexing repetitive sequences Theor Comput Sci 2013 483 115 133

[B67] Gagie T Gawrychowski P Kärkkäinen J Nekrich Y Puglisi SJ A faster grammar-based self-index Proceedings of the 6th International Conference on Language and Automata Theory and Applications (LATA) 2012 Springer-Verlag, Berlin-Heidelberg: LNCS 7183 240 251

[B68] Do HH Jansson J Sadakane K Sung W-K Fast relative Lempel-Ziv self-index for similar sequences Proceedings of the Joint International Conference on Frontiers in Algorithmics and Algorithmic Aspects in Information and Management (FAW-AAIM) 2012 Springer-Verlag, Berlin-Heidelberg: LNCS 7285 291 302

[B69] Gagie T Gawrychowski P Puglisi SJ Faster approximate pattern matching in compressed repetitive texts Proceedings of the 22nd International Symposium on Algorithms and Computation (ISAAC) 2011 Springer-Verlag, Berlin-Heidelberg: LNCS 7074 653 662

[B70] Ahmadi A Behm A Honnalli N Li C Weng L Xie X Hobbes: optimized gram-based methods for efficient read alignment Nucleic Acids Res 2012 40 6 e41 22199254 10.1093/nar/gkr1246 PMC3315303

[B71] Langmead B Trapnell C Pop M Salzberg SL Ultrafast and memory-efficient alignment of short DNA sequences to the human genome Genome Biol 2009 10 3 R25 19261174 10.1186/gb-2009-10-3-r25 PMC2690996

[B72] Langmead B Salzberg SL Fast gapped-read alignment with Bowtie Nature Methods 2012 9 357 359 22388286 10.1038/nmeth.1923 PMC3322381

[B73] Li H Durbin R Fast and accurate short read alignment with Burrows–Wheeler transform Bioinformatics 2009 25 14 1754 1760 19451168 10.1093/bioinformatics/btp324 PMC2705234

[B74] Li H Durbin R Fast and accurate long-read alignment with Burrows–Wheeler transform Bioinformatics 2010 26 5 589 595 20080505 10.1093/bioinformatics/btp698 PMC2828108

[B75] Li R Yu C Li Y Lam T-W Yiu S-M Kristiansen K Wang J SOAP2: an improved ultrafast tool for short read alignment Bioinformatics 2009 25 15 1966 1967 19497933 10.1093/bioinformatics/btp336

[B76] Marco-Sola S Sammeth M Guigó R Ribeca P The GEM mapper: fast, accurate and versatile alignment by filtration Nat Methods 2012 9 12 1185 1188 23103880 10.1038/nmeth.2221

[B77] Kärkkäinen J Fast BWT in small space by blockwise suffix sorting Theor Comput Sci 2007 387 249 257

[B78] Ferragina P Gagie T Manzini G Lightweight data indexing and compression in external memory Algorithmica 2012 63 3 707 730

[B79] Wang Z Gerstein M Snyder M RNA-Seq: a revolutionary tool for transcriptomics Nat Rev Genet 2009 10 1 57 63 19015660 10.1038/nrg2484 PMC2949280

[B80] Trapnell C Pachter L Salzberg SL TopHat: discovering splice junctions with RNA-Seq Bioinformatics 2009 25 9 1105 1111 19289445 10.1093/bioinformatics/btp120 PMC2672628

[B81] Rivals E CRAC: an integrated approach to the analysis of RNA-seq reads Genome Biol 2013 14 3 R30 23537109 10.1186/gb-2013-14-3-r30 PMC4053775

[B82] Alamancos GP Agirre E Eyras E Methods to study splicing from high-throughput RNA Sequencing data Publicly available preprint arXiv:1304.5952v1 10.1007/978-1-62703-980-2_26 24549677

[B83] Li H Exploring single-sample SNP and INDEL calling with whole-genome de novo assembly Bioinformatics 2012 28 14 1838 1844 22569178 10.1093/bioinformatics/bts280 PMC3389770

[B84] Liu C-M Wong TKF Wu E Luo R Yiu S-M Li Y Wang B Yu C Chu X Zhao K Li R Lam TW SOAP3: ultra-fast GPU-based parallel alignment tool for short reads Bioinformatics 2012 28 6 878 879 22285832 10.1093/bioinformatics/bts061

[B85] Luo R Wong T Zhu J Liu C-M Zhu X Wu E Lee L-K Lin H Zhu W Cheung DW Ting H-F Yiu S-M Peng S Yu C Li Y Li R Lam TW SOAP3-dp: Fast, accurate and sensitive GPU-based short read aligner PLoS ONE 2013 8 5 e65632 23741504 10.1371/journal.pone.0065632 PMC3669295

[B86] Gog S Petri M Optimized succinct data structures for massive data Softw Pract Exp 2013 doi: 10.1002/spe.2198

[B87] Loh P-R Baym M Berger B Compressive genomics Nat Biotechnol 2012 30 7 627 630 22781691 10.1038/nbt.2241

[B88] Altschul SF Gish W Miller W Myers EW Lipman DJ Basic local alignment search tool J Mol Biol 1990 215 3 403 410 2231712 10.1016/S0022-2836(05)80360-2

[B89] Kent WJ BLAT–the BLAST-like alignment tool Genome Res 2002 12 4 656 664 11932250 10.1101/gr.229202 PMC187518

[B90] Deorowicz S Debudaj-Grabysz A Grabowski Sz Disk-based k-mer counting on a PC BMC Bioinformatics 2013 14 Article no. 160 10.1186/1471-2105-14-160 PMC3680041 23679007

[B91] Zerbino DR Birney E Velvet: algorithms for de novo short read assembly using de Bruijn graphs Genome Res 2008 18 5 821 829 18349386 10.1101/gr.074492.107 PMC2336801

[B92] Simpson JT Wong K Jackman SD Schein JE Jones SJM Birol I ABySS: A parallel assembler for short read sequence data Genome Res 2009 19 6 1117 1123 19251739 10.1101/gr.089532.108 PMC2694472

[B93] Cao MD Dix TI Allison L A genome alignment algorithm based on compression BMC Bioinformatics 2010 11 1 599 21159205 10.1186/1471-2105-11-599 PMC3022628

[B94] Bhaduri A Qu K Lee CS Ungewickell A Khavari P Rapid identification of nonhuman sequences in high throughput sequencing data sets Bioinformatics 2012 28 8 1174 1175 22377895 10.1093/bioinformatics/bts100 PMC3324519

[B95] Ferragina P Giancarlo R Greco V Manzini G Valiente G Compression-based classification of biological sequences and structures via the universal similarity metric: experimental assessment BMC Bioinformatics 2007 8 252 17629909 10.1186/1471-2105-8-252 PMC1939857

[B96] Li M Chen X Li X Ma B Vitányi PMB The similarity metric IEEE Trans Inf Theory 2004 50 12 3250 3264

[B97] Freschi V Bogliolo A A lossy compression technique enabling duplication-aware sequence alignment Evol Bioinformatics 2012 8 171 180 10.4137/EBO.S9131 PMC3327517 22518086

[B98] Illumina HiSeq 2500 system user guide 2012 [http://supportres.illumina.com/documents/myillumina/223bf628-0b46-409f-aa3d-4f3495fe4f69/hiseq2500_ug_15035786_ a_public.pdf]

[B99] Illumina New algorithms increase computing efficiency for IGN whole-genome analysis 2013 [http://res.illumina.com/documents/products/technotes/technote_ign_isaac_software.pdf]

