# Supplementary material for: Data compression for sequencing data
Source: Algorithms Mol Biol. 2013 Nov 19;8:25. doi: 10.1186/1748-7188-8-25 (PMC3868316; doi:10.1186/1748-7188-8-25)
Supplement: Additional file 1 — Description of selected compression methods. [file 1748-7188-8-25-S1.pdf]

Additional file

Statistical coding

The need for compact representation of data, that is, their compression, was observed centuries ago but the first widely known applications appeared in the first half of the 19th century. Louis Braille designed his scheme to enable the blind to read printed texts. The Braille code defines a 3×2 dots matrix and each of 26 letters is assigned a combination of the dots. Since there are fewer basic textual characters (including digits and punctuation marks) than the number of possible dot combinations, $2^6 = 64$, some of them were initially unassigned. This solution allowed encoding the most common words or series of letters as single dot combinations, being an example of *dictionary compression*.

The Morse code, used for radio communication, was developed by Samuel Morse and Alfred Vail. It assigns a variable-length sequence of dots and dashes to each letter and digit. The frequent symbols are represented by shorter codewords, the rare ones—by longer codewords. This is an example of what is known as a *statistical compression* method, which uses variable-length codewords instead of fixed-length ones (like ASCII or Unicode symbol codewords used in computers to store texts). Roughly speaking, the reduction of the data size is because the gains obtained for frequent symbols overtake the loss for the rare ones.

The statistical and dictionary methods are the fundamentals of modern data compression algorithms. The first formalized approach for statistical methods was made by Claude E. Shannon in 1948, in which his famous paper on theory of communication and data compression was published. Shannon derived a formula that to obtain the best possible compression each alphabet symbol should be encoded as a sequence of bits of length $-\log_2 p_i$, where p_i is the probability of the symbol appearance. The first code satisfying this rule was proposed three years later by 26-year-old student David Huffman in his term paper, given to him by his professor, Robert Fano. The Huffman code is still used as a component of popular contemporary compression methods (Fig. 2).

(a) Input sequence:

baffdcafecafbfafddf

(b) Histogram:

Symbol	a	b	c	d	e	f
No. of occ.	4	2	2	3	1	7

(c) Huffman tree:

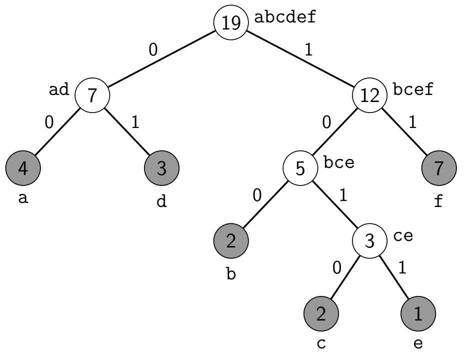

(d) Codewords:

a – 00	b – 100	c – 1010
d – 01	e – 1011	f – 11

(e) Huffman-encoded sequence:

1000011110110100011101110100011100110011010111

b a f f d c a f e c a f b f a f d d f

Figure 2: Huffman coding looks at individual symbols, ignoring their context. What matters are their frequencies: in accordance with the golden rule of compression, it assigns shorter codewords to more frequent symbols and longer codewords to rarer symbols. The tricky part, solved by Huffman, is how to do it in detail. The input sequence, over an alphabet of size 6, is given in (**a**). First, the counts of symbols' frequencies are found (see (**b**)). Then, a binary tree, with its leaves representing the binary codewords assigned to particular symbols, is built stepwise.

What we have at the beginning are 6 leaf nodes, one per each alphabet symbol. They are not connected yet into a tree. The numbers in the nodes (see (**c**)) correspond to their weights, and for the leaves they are just the symbols' frequencies. First, the two least frequent (least weighted) symbols are found, c and e in our example. One of them, say, c, obtains binary digit 0, while the other, e, obtains binary digit 1. These two rarest symbols are merged into one supersymbol, ce, and a tree's internal node merging the leaves for c and e is created, with its weight being the sum of the merged nodes. After that, the whole procedure is repeated, i.e., now the two least frequent symbols are b and ce. Note that with each merge the number of remaining symbols (including supersymbols) is decreased by one. Continuing this way, we finally obtain only one supersymbol, representing the whole alphabet, which equivalently produces the tree's root. Each path from the root (see (**c**)) to a leaf stores the binary codeword for an alphabet symbol (e.g., for symbol b it is 100 in our example). The whole mapping is presented in (**d**). The resulting *Huffman tree* can be shown to represent an optimal encoding for given symbols' frequencies; in other words, for a given alphabet and the symbol frequencies, no other code based only on those frequencies can yield better compression. The concatenation of obtained codewords for the symbols in their input sequence is the resulting compressed message (see (**e**)). It is easy to notice that Huffman coding is *instantaneous*, that is, a decoder is immediately capable of finding the boundaries between the (variable-sized) codewords. This results from the fact that Huffman coding is *prefix*: no codeword is a prefix of another, since no path from the root to a leaf can be a beginning of another path (to another leaf).

Burrows–Wheeler transform

In 1994 Mike Burrows and David Wheeler published a surprising compression algorithm based on the transform which was soon named after them, i.e., is now known as the Burrows–Wheeler transform (BWT). The BWT permutes the input symbols in a reversible way. The order of symbols is dictated by the lexicographical order of the suffixes of the input sequence that follow those symbols (see Fig. 3). In this way, for compressible data the same symbols tend to reappear close to each other, or even long runs of one symbol are formed, which is beneficial for further compression.

This is not everything though. A few years later, in 2000, Paolo Ferragina and Giovanni Manzini showed an amazing way to search for a pattern directly in the BWT output[56]. This mechanism, sometimes called *LF-mapping* (where L and F stand for the last and the first column of the conceptual matrix of suffixes), is the basis of their *FM-index* (see Fig. 4). The FM-index is very popular in bioinformatics, in particular for read alignment onto a reference genome. Its popularity comes from the stark contract between traditional text indexes, like the suffix array, which need (at least) 5 bytes per input symbol, and this novel paradigm of *compressed* (or: *succinct*) text indexes, needing basically not much more than 2 bits per symbol for DNA, i.e., about 15 times less memory. Although sometimes compressed data structure trade some space for faster search, the difference in space requirement remains very significant.

(a)

A	G	G	A	T	A	C	A	G	G	C	A	A	G	$

(b)

A	G	G	A	T	A	C	A	G	G	C	A	A	G	$
G	G	A	T	A	C	A	G	G	C	A	A	G	$	A
G	A	T	A	C	A	G	G	C	A	A	G	$	A	G
A	T	A	C	A	G	G	C	A	A	G	$	A	G	G
T	A	C	A	G	G	C	A	A	G	$	A	G	G	A
A	C	A	G	G	C	A	A	G	$	A	G	G	A	T
C	A	G	G	C	A	A	G	$	A	G	G	A	T	A
A	G	G	C	A	A	G	$	A	G	G	A	T	A	C
G	G	C	A	A	G	$	A	G	G	A	T	A	C	A
G	C	A	A	G	$	A	G	G	A	T	A	C	A	G
C	A	A	G	$	A	G	G	A	T	A	C	A	G	G
A	A	G	$	A	G	G	A	T	A	C	A	G	G	C
A	G	$	A	G	G	A	T	A	C	A	G	G	C	A
G	$	A	G	G	A	T	A	C	A	G	G	C	A	A
$	A	G	G	A	T	A	C	A	G	G	C	A	A	G

(c)

A	A	G	$	A	G	G	A	T	A	C	A	G	G	C
A	C	A	G	G	C	A	A	G	$	A	G	G	A	T
A	G	G	A	T	A	C	A	G	G	C	A	A	G	$
A	G	G	C	A	A	G	$	A	G	G	A	T	A	C
A	G	$	A	G	G	A	T	A	C	A	G	G	C	A
A	T	A	C	A	G	G	C	A	A	G	$	A	G	G
C	A	A	G	$	A	G	G	A	T	A	C	A	G	G
C	A	G	G	C	A	A	G	$	A	G	G	A	T	A
G	A	T	A	C	A	G	G	C	A	A	G	$	A	G
G	C	A	A	G	$	A	G	G	A	T	A	C	A	G
G	G	A	T	A	C	A	G	G	C	A	A	G	$	A
G	G	C	A	A	G	$	A	G	G	A	T	A	C	A
G	$	A	G	G	A	T	A	C	A	G	G	C	A	A
T	A	C	A	G	G	C	A	A	G	$	A	G	G	A
$	A	G	G	A	T	A	C	A	G	G	C	A	A	G

(d)

C	T	$	C	A	G	G	A	G	G	A	A	A	A	G

Figure 3: The Burrows–Wheeler transform. (a) The input sequence $\mathcal{T}$, terminated with $\$$, an artificial symbol, lexicographically greatest in the alphabet. (b) All suffixes of $\mathcal{T}$ in natural (left-to-right) order. They form rows of a conceptual matrix. In a computer, the suffixes are represented as their start locations in $\mathcal{T}$. (c) Lexicographically sorted suffixes. BWT-based algorithms need the first and the last column of the resulting matrix. Note that the first column can be represented very succinctly as the locations where the symbol changes. There are only $\sigma + 1$ such locations, where σ is the alphabet size (e.g., 4). (d) The last column, which is $BWT(\mathcal{T})$.

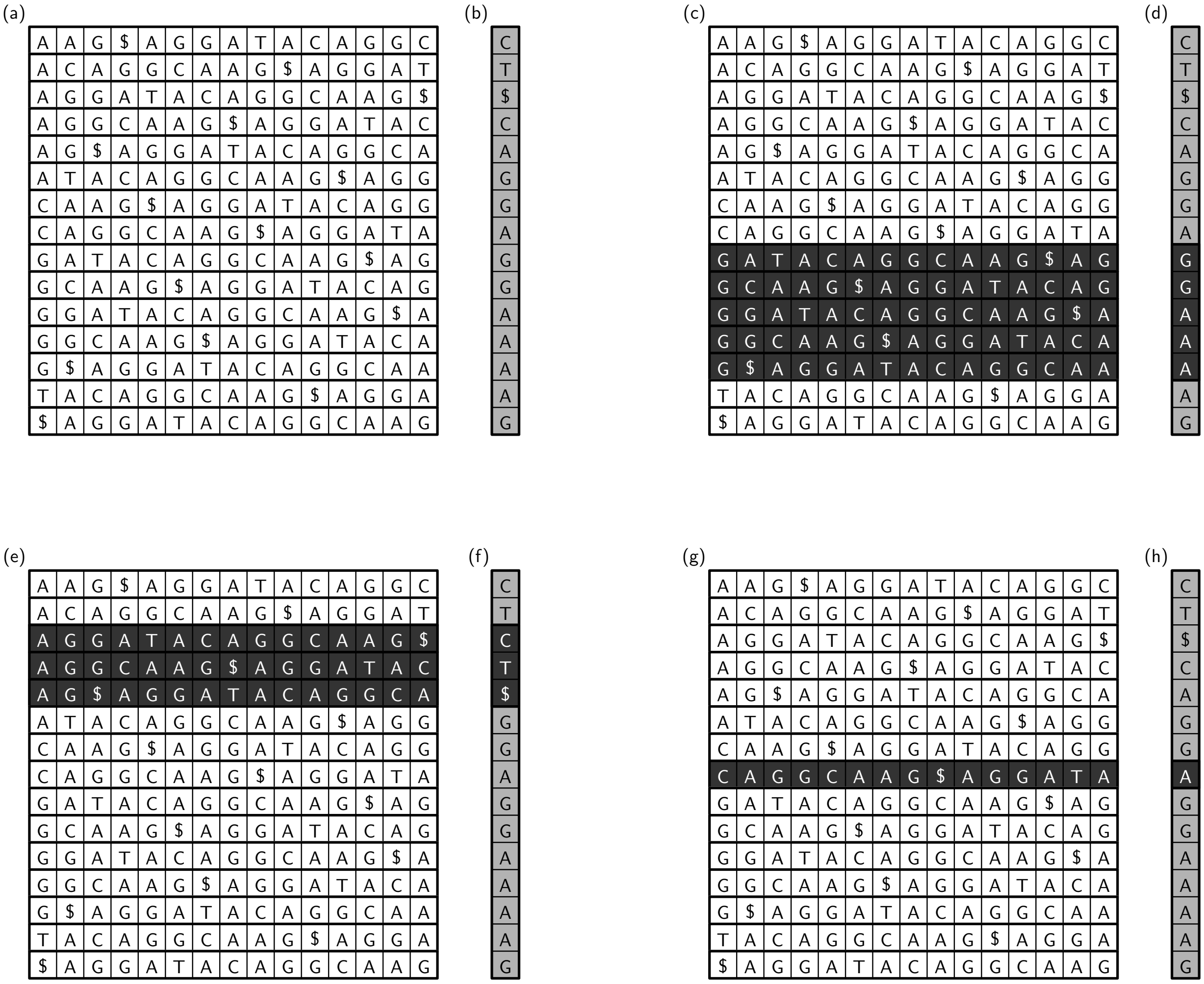

Figure 4: The FM-index search mechanism. We look for pattern CAG. (a) The BWT conceptual matrix. (b) The last column. (c) and (d) The result of the search for the last symbol of the pattern, G. The corresponding rows and the interval in the last column are darkened. (e) and (f) The next-to-last symbol of the pattern, A, in the locations where it prepends symbol G, is sought for. The result is an interval of rows starting with AG. As the suffixes are sorted, all suffixes starting with AG must be in a contiguous area of the matrix. (g) and (h) Finally, the symbol C is sought for, and the result is the range of rows (of cardinality 1) starting with CAG. The search is over. To tell the locations of the found patterns (apart from merely their count), some additional data structures are needed.
